# Socioeconomic disparities in alcohol-related depression: a national cohort study of low-income medical aid beneficiaries and national health insurance beneficiaries in Korea

**DOI:** 10.1186/s12889-024-19665-6

**Published:** 2024-08-13

**Authors:** Su Kyoung Lee, Yong Jin Kwon

**Affiliations:** 1 Institute of Health and Environment Graduate School of Public, Health Seoul National University, Seoul, Republic of Korea; 2 Department of Public Healthcare Center, Seoul National University Hospital, Daehak-Ro Jongno-Gu, 101, 03080 Seoul, Republic of Korea

**Keywords:** Alcohol consumption, Depression, Socioeconomic status

## Abstract

**Objective:**

To examine the association between patterns of alcohol consumption in the past and the risk of depression among medical aid beneficiaries and National Health Insurance beneficiaries in Korea.

**Methods:**

We used data from the National Health Information Database (NHID) of 1,292,618 participants who underwent health checkups in 2015–16 and 2017–18. We categorized alcohol consumption into four groups: continuous high, increased, decreased, and non-consumers. We followed the participants from 2019 to 2021 and identified new episodes of depression. We calculated adjusted odds ratios (aOR) and 95% confidence intervals (CI) for depression by alcohol consumption groups and socioeconomic status.

**Results:**

Medical aid beneficiaries had higher risks of depression than National Health Insurance beneficiaries across all alcohol consumption groups. The highest risk was observed among continuous high consumers (aOR, 2.31; 95% CI, 1.36–3.93), followed by increased (aOR, 1.51; 95% CI, 1.17–1.94), decreased (aOR, 1.48; 95% CI, 1.18–1.84), and non-consumers (aOR, 1.37; 95% CI, 1.22–1.54).

**Conclusions:**

Socioeconomic status and patterns of alcohol consumption in the past are associated with the risk of depression. Public health interventions should consider both factors to reduce alcohol-related depression and health inequalities.

**Supplementary Information:**

The online version contains supplementary material available at 10.1186/s12889-024-19665-6.

## Introduction

In 2019, mental disorders accounted for 16% of global DALYs, equating to 418 million disability-adjusted life years (DALYs) [1]. Depression, affecting over 280 million globally, was among the top 25 global health burdens before 2020 [2, 3]. Depression is also a leading cause of disability and mortality, as it increases the risk of suicide and other physical illnesses [2]. Therefore, it is important to identify and manage the risk factors that are associated with depression. One of them could be alcohol consumption, which is a cause of over 200 diseases, injuries, and other health conditions [4]. Alcohol consumption is influenced by various factors, such as socioeconomic status (SES), which also affects the health and social outcomes of alcohol use [5]. Growing evidence suggests that alcohol consumption is linked to depression and that this association is stronger among disadvantaged social groups, who experience more alcohol-related harm even after adjusting for various confounders [6, 7].

Observational and clinical studies have consistently shown that alcohol consumption can lead to negative social, psychological, and physical outcomes, including an increased risk of depression [8, 9]. Nonetheless, there is an evident paradox in alcohol-related harm [10], revealing a notable disparity between alcohol consumption rates and the incidence of documented alcohol-related harm across different social groups[11]. Findings from a cohort study, tracking around 50,000 participants across approximately 420,000 person-years, indicated that heightened alcohol consumption led to harm across all demographics, yet individuals with lower socioeconomic status suffered more than three times the alcohol-related harm compared to other groups[6].

In South Korea, health disparities between Medical Aid beneficiaries and National Health Insurance (NHI) beneficiaries are well-documented [12]. Medical Aid beneficiaries, who are typically from lower socioeconomic backgrounds, report poorer health-related quality of life and higher rates of mental health issues, including depression, compared to NHI beneficiaries[13] Moreover, Drinking alcohol in South Korea has been shown to be associated with a range of negative health and social outcomes, including physical illness, mental health problems, and social problems such as domestic violence and crime. The effects of these harms are disproportionately greater in certain social groups, which can exacerbate existing inequalities [14]. Although substantial research exists on the relationship between alcohol use, depressive symptoms, and socioeconomic status (SES) individually, there is a paucity of research examining disparities in depression risk by alcohol consumption between groups of different SES.

To bridge this evidence gap, we investigated the association between changes in previous alcohol consumption patterns and the risk of depression. This investigation was conducted using a population-based study that included Medical Aid beneficiaries—individuals from socioeconomically vulnerable groups selected from a customized National Health Information Database (NHID).

## Methods

### Study design

This study is a cohort study with a nested case–control design. We started with a defined population of medical aid beneficiaries and National Health Insurance beneficiaries. These individuals were followed over time from January 1, 2019, to December 31, 2021, to identify new cases of depression. Within this cohort, we conducted a nested case–control analysis, comparing cases of medical aid beneficiaries with control cases of National Health Insurance beneficiaries during the follow-up period. We then compared the incidence of new depression between these cases and controls, which was our primary outcome of interest.

### Study population

Data for this study were extracted from a customized National Health Information Database (NHID) provided by the National Health Insurance Service (NHIS) in Korea. The NHIS provides health insurance services to approximately 97% of the Korean population. The NHID data cover all Medical Aid beneficiaries in South Korea, excluding foreigners. The data source used in this study, customized claims data from the NHID, is census data, which strengthens the representativeness and reliability of the study. South Korea has a separate health coverage system for low-income people, but all of their medical information is collected by the National Health Insurance (NHI) corporation. In this study, Medical Aid beneficiaries were identified as individuals who became eligible for medical benefits at the beginning of the years 2017 and 2018 following the Medical Care Assistance Act [15]. National Health Insurance beneficiaries were defined as participants who did not receive medical benefits until 2018.

The Medical Aid Program in South Korea is a public assistance initiative that provides comprehensive healthcare benefits to low-income people. Operating separately from the National Health Insurance (NHI) system, the program covers a wide range of medical expenses, including outpatient care, inpatient care, and medication [16]. Beneficiaries are generally those who fall below a certain income threshold (40% of median income). The program categorizes beneficiaries into two types: Medical Aid Type 1 (for those who are incapable of working) and Medical Aid Type 2 (for those who are capable of working) [12]. The Medical Aid program not only covers most medical expenses but also provides free or low-cost preventive care services. Medical Aid beneficiaries and National Health Insurance beneficiaries are distinct groups that do not overlap. Health insurance claims, including medical health screening results, treatments, and prescribed medications, were collected by the NHIS.

**Fig. 1:**
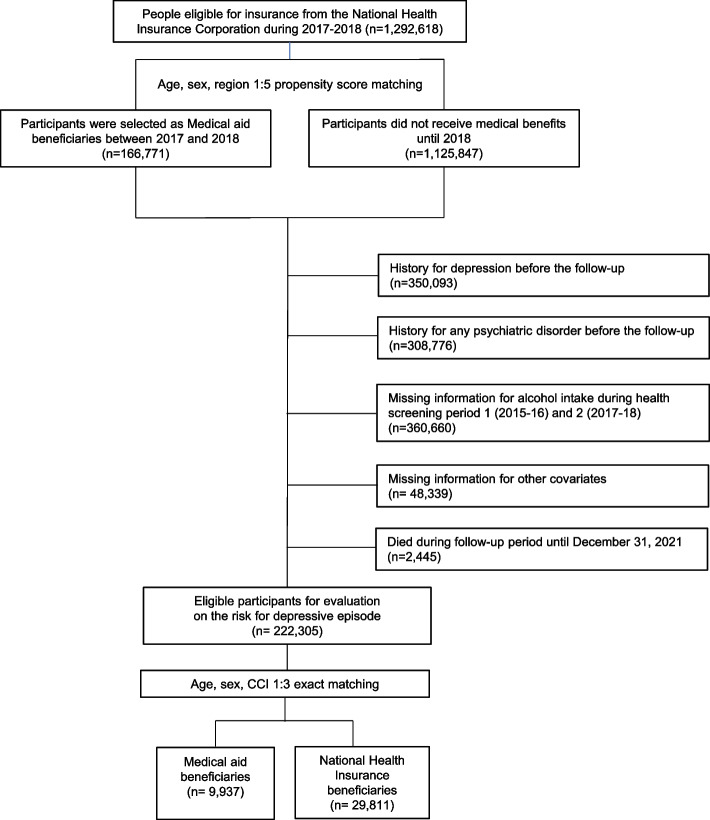
Flow diagram for the inclusion of the study population

In this study, a total of 1,292,618 participants were initially enrolled, with those who transitioned to medical aid beneficiaries between 2017 and 2018 comprising the case group, and the control group was matched at a 1:5 ratio using age, sex, and regional propensity scores. It included the medical aid beneficiaries' case population, who became medical aid beneficiaries between 2017 and 2018 (n = 166,771), as well as control participants who did not receive medical benefits until 2018 (n = 1,125,847). Before the follow-up point on January 1, 2019, individuals with a history of depression (defined as medication or diagnostic code F32-33) were excluded (n = 350,093). Those with other mental health conditions (ICD-10 F codes) were also excluded (n = 308,776). Individuals without alcohol survey data during the health checkups in 2015–16 (period 1) and 2017–18 period 2) were excluded as well (n = 360,660). Furthermore, individuals without data on other covariates (n = 48,339) or who died during the follow-up period were excluded(n = 2,445). Therefore, eligible participants for evaluation of the risk for depressive episodes (n = 170,448). Finally, to minimize statistical bias, we implemented 1:3 exact matching based on age, sex, and Charlson Comorbidity Index (CCI) to align the distribution of medical aid beneficiaries and National Health Insurance beneficiaries. A total of 9,937 medical aid beneficiaries and 29,811 National Health Insurance beneficiaries were enrolled and followed up from January 1, 2019, to December 31, 2021, to monitor depression events. (Fig. 1). The Institutional Review Board of Seoul National University Hospital Biomedical Research Institute approved this study (No.: E-2301–049-1394). Informed consent was waived because the database was provided for research purposes in an anonymized form under strict confidentiality guidelines.

### Exposure

The level of alcohol consumption was determined based on the frequency of alcohol intake per week, measured during two consecutive health checkups conducted between 2015–16 (period 1) and 2017–18 (period 2). The frequency pattern between periods was categorized into four groups: (1) no alcohol consumption for both periods, (2) increased alcohol consumption from period 1 to 2, (3) decreased alcohol consumption from period 1 to 2, and (4) continuously high alcohol consumption for both periods (≥ 5 times per week).

### Outcome

The primary outcome of this study was the incident depression during the follow-up period (from January 1, 2019 to December 31, 2021), as defined by the International Classification of Diseases, Tenth Revision (ICD-10) codes F32-F33 (major depressive disorder with a single episode and major depressive disorder with recurrent episodes), and prescription records for antidepressants. These ICD-10 codes for depression and suicide attempts were adopted from a previous study [17].

### Covariates

The following variables were considered as key variables for multivariable analyses: age, sex, household income, body mass index (BMI), cigarette smoking, moderate-to-vigorous physical activity (MVPA), history of hypertension, diabetes mellitus, dyslipidemia, and Charlson comorbidity index (CCI). These variables were obtained from the National Health Insurance Service (NHIS) database. Household income was categorized as quartiles based on the insurance premium. BMI was categorized into four groups < 18.5, 18.5–23, 23–25, and ≥ 25. Smoking was categorized as current, former, and never smoker. MVPA was assessed by the frequency of weekly MVPA using self-reported questionnaires from the NHIS health screening program. By adding the frequency per week (days per week) for moderate and vigorous physical activity, we categorized it into 4 levels: physically inactive; 1–2 times/week; 3–4 times/week; and ≥ 5 times/week. Underlying comorbidities were assessed by the CCI score using claims data before the follow-up validated in the previous study [18]. Diagnosis of hypertension, diabetes mellitus, and dyslipidemia was confirmed by the laboratory results [hypertension: (systolic blood pressure ≥ 140mmhg or diastolic blood pressure ≥ 90mmhg), diabetes: fasting blood glucose ≥ 126 mg/dL, dyslipidemia: total cholesterol ≥ 250 mg/dl] or relevant medication usage reported at the health screening, both of which were obtained from the NHIS health check-up data.

### Statistical analysis

We analyzed the risk association with depression among medical aid beneficiaries and National Health Insurance beneficiaries based on alcohol consumption patterns. We followed up to see if there were any new cases of depression from January 1, 2019, to December 31, 2021. The number of events for the results was presented as n (%) mean ± standard deviation (SD) for continuous variables and n (%) for categorical variables. The number of events for the results was expressed as n (%). Adjusted odds ratios (aOR) and 95% confidence intervals (CI) were calculated using multivariate logistic regression. Initially, we adjusted for age and gender for multivariate regression analysis. After this initial adjustment, We further adjusted for other potential confounding factors, including income rank, BMI, smoking status, physical activity, history of hypertension, diabetes, dyslipidemia, and CCI. The CCI score, widely used as a measure of multiple comorbidities, was used to assess the history of hypertension, diabetes, and dyslipidemia through risk adjustment, laboratory results, or associated medication use reported during health examinations. For the subgroup analyses, we stratified the patients by age (< 65, ≥ 65), sex (men, women), comorbidity (CCI; 0, 1, ≥ 2), hypertension (yes, no), diabetes mellitus (yes, no), and dyslipidemia (yes, no). P-values less than 0.05 were considered statistically significant in a two-sided manner. All data collection, mining, and statistical analyses were performed using SAS 9.4 (SAS Institute, Cary, NC).

## Results

The analysis cohort consisted of a total of 222,305 participants, including cases (n = 9,937) who switched to medical aid beneficiaries in 2017 and 2018, matched in a 1:3 ratio to form a control group (n = 29,811). The alcohol consumption patterns between period 1 and period 2 displayed the following findings: (1) Constant non-drinkers numbered 4,400 individuals (44.3%) among medical aid beneficiaries and 12,671 individuals (42.5%) among National Health Insurance beneficiaries; (2) Those who increased their drinking habits amounted to 1,095 individuals (11.0%) among medical aid beneficiaries and 3,671 individuals (12.3%) among National Health Insurance beneficiaries; (3) Decreased drinkers included 4,234 individuals (42.6%) among medical aid beneficiaries and 12,881 individuals (43.2%) among National Health Insurance beneficiaries; and (4) Individuals consistently consuming heavily accounted for 208 individuals (2.1%) among medical aid beneficiaries and 588 individuals (2.0%) among National Health Insurance beneficiaries. The distribution of demographic characteristics between the case and control groups appeared generally similar (Table 1).

**Table 1:** Descriptive characteristics of the study participants

**Variables**	**Medical Aid Beneficiaries**^**a**^ (n = 9937)	**National Health Insurance beneficiaries**^**b**^ (n = 29,811)
**Age, years**	54.5 (13.5)	54.5 (13.5)
**Sex, n (%)**		
Male	6020 (60.6)	18,060 (60.6)
Female	3917 (39.4)	11,751 (39.4)
**Alcohol consumption (period 1), n (%)**		
0 days/week	4992 (50.2)	14,687 (49.3)
1–2 days/week	3496 (35.2)	10,713 (35.9)
3–4 days/week	1019 (23.9)	3247 (10.9)
≥ 5 days/week	430 (4.3)	1164 (3.9)
**Alcohol consumption (period 2), n (%)**		
0 days/week	5288 (53.2)	14,977 (50.2)
1–2 days/week	3266 (32.9)	10,428 (35.0)
3–4 days/week	994 (10.0)	3201 (10.7)
≥ 5 days/week	389 (3.9)	1205 (4.0)
**Alcohol consumption pattern from period 1 to period 2, n (%)**		
No alcohol consumption	4400 (44.3)	12,671 (42.5)
Increased alcohol consumption	1095 (11.0)	3671 (12.3)
Decreased alcohol consumption	4234 (42.6)	12,881 (43.2)
Continuously high alcohol consumption	208 (2.1)	588 (2.0)
**Household income, n (%)**		
1st quartile	3539 (35.6)	6065 (20.3)
2nd quartile	1717 (17.3)	5814 (19.5)
3rd quartile	2075 (20.9)	7836 (26.3)
4rd quartile (highest)	2606 (26.2)	10,096 (33.9)
**Baseline comorbidities, n (%)**		
Hypertension	4298 (43.3)	12,079 (40.5)
Diabetes	1662 (16.7)	4838 (16.2)
Dyslipidemia	3172 (31.9)	9256 (31.1)
**Charlson comorbidity index, n (%)**		
0	5212 (52.5)	15,636 (52.5)
1	2558 (25.7)	7674 (25.7)
≥ 2	2167 (21.8)	6501 (21.8)
**Body mass index, n (%)**		
< 18.5 kg/m2	273 (2.8)	644 (2.2)
18.5–23.0 kg/m2	3182 (32.0)	9542 (32.0)
23.0–25.0 kg/m2	2478 (24.9)	7544 (25.3)
≥ 25.0 kg/m2	4004 (40.3)	12,081 (40.5)
**Moderate-to-vigorous physical activity, n (%)**		
0 days/week	4069 (41.0)	11,705 (39.3)
1–2 days/week	1726 (17.4)	5442 (18.3)
3–4 days/week	1691 (17.0)	5066 (17.0)
≥ 5 days/week	2451 (24.7)	7598 (25.5)
**Cigarette smoking, n (%)**		
Non-smoker	5395 (54.3)	16,726 (56.1)
Former smoker	2254 (22.7)	6723 (22.6)
Current smoker	2288 (23.0)	6362 (21.3)

Continuous variables were presented as mean ± standard deviation (SD) and categorical variables as n (%)

The level of alcohol consumption was determined based on the frequency of alcohol intake per week, measured during two consecutive health checkups conducted between 2015–16 (period 1) and 2017–18 (period 2). The frequency pattern between periods was categorized into four groups: (1) no alcohol consumption for both periods, (2) increased alcohol consumption from period 1 to 2, (3) decreased alcohol consumption from period 1 to 2, and (4) continuously high alcohol consumption for both periods (≥ 5 times per week). Depression defines by any antidepressant medication intake or diagnosed by expert physician (ICD-10 F32, F33)

^a^Medical Aid Beneficiaries were defined by Participants were selected as Medical Aid Beneficiaries for the first time in their lives between 2017 and 2018

^b^National Health Insurance beneficiaries were define by Participants did not receive medical benefits until 2018

### Association of changes in alcohol consumption with the risk of depression

The relationship between the risk of depression based on changes in alcohol consumption is presented in Table 2, categorized by the periods of 2015–2016 (Period 1) and 2017–2018 (Period 2). Throughout both periods, individuals receiving medical benefits consistently exhibited an elevated risk of depression compared to those with health insurance, regardless of their alcohol consumption levels. Nonetheless, there was a discernible trend indicating that the odds ratio (OR) for depression risk increased with higher alcohol consumption. This suggests a trend where the disparity in the risk of depression incidents between the two groups widened as the weekly alcohol consumption frequency increased.

**Table 2:** Association of Changes in Alcohol Consumption with Depression Risk among Medical Aid Beneficiaries and National Health Insurance beneficiaries

**Alcohol consumption per week**	**Multivariable-adjusted OR (95% CI)**		*P* value
	**Medical Aid Beneficiaries**^**a**^	**National Health Insurance beneficiaries**^**b**^	
**Period 1 (2015–16)**			
0 days	1.39 (1.24–1.55)	1.00 (ref)	< .001
1–2 days	1.36 (1.16–1.60)	1.00 (ref)	< .001
3–4 days	1.44 (1.09–1.89)	1.00 (ref)	0.010
≥ 5 days	1.95 (1.35–2.82)	1.00 (ref)	< .001
**Period 2 (2017–18)**			
0 days	1.38 (1.24–1.54)	1.00 (ref)	< .001
1–2 days	1.38 (1.17–1.63)	1.00 (ref)	< .001
3–4 days	1.42 (1.06–1.89)	1.00 (ref)	0.020
≥ 5 days	2.02 (1.38–2.95)	1.00 (ref)	< .001

The level of alcohol consumption was determined based on the frequency of alcohol intake per week, measured during two consecutive health checkups conducted between 2015–16 (period 1) and 2017–18 (period 2). Depression defines by any antidepressant medication intake or diagnosed by expert physician (ICD-10 F32, F33)

aOR was calculated using multivariate adjusted logistic regression and presented with 95% CI. Adjusted by age, sex, household income, baseline comorbidities (hypertension, diabetes, dyslipidemia), cigarette smoking, body mass index, moderate-to-vigorous physical activity, and Charlson Comorbidity Index

^a^Medical Aid Beneficiaries were defined by Participants were selected as Medical Aid Beneficiaries for the first time in their lives between 2017 and 2018

^b^National Health Insurance beneficiaries were define by Participants did not receive medical benefits until 2018

For individuals consuming alcohol more than five days a week, medical aid beneficiaries showed nearly double the risk of depression incidents compared to National Health Insurance beneficiaries in both periods(Period 1: aOR, 1.95; 95% CI, 1.35–2.83, Period 2: aOR, 2.02; 95% CI, 1.38–2.95). Similarly, those consuming alcohol three to four days a week had approximately 1.4 times higher risk(Period 1: aOR, 1.44; 95% CI, 1.09–1.89, Period 2: aOR, 1.42; 95% CI, 1.06–1.89), and individuals consuming alcohol one to two days a week exhibited a 1.36 times higher risk among medical aid beneficiaries compared to National Health Insurance beneficiaries (Period 1: aOR, 1.36; 95% CI, 1.16–1.60, Period 2: aOR, 1.38; 95% CI, 1.17–1.63). For those abstaining from alcohol, the risk was nearly similar, ranging from 1.38 times higher for medical aid beneficiaries in both periods compared to National Health Insurance beneficiaries (Period 1: aOR, 1.39; 95% CI, 1.24–1.55, Period 2: aOR, 1.42; 95% CI, 1.24–1.54).

Table 3 demonstrates the association between changes in alcohol intake and the risk of depressive episodes. The study conducted two models of analysis: one adjusted for sex and age (model 1), and another further adjusted for various factors such as household income, comorbidities, lifestyle habits, and health status (model 2). Both models consistently demonstrated that individuals under medical aid had a consistently higher risk of experiencing depressive episodes compared to those with health insurance, regardless of changes in alcohol intake. However, concerning fluctuations in alcohol consumption, this risk discrepancy between medical aid beneficiaries and National Health Insurance beneficiaries expanded.

**Table 3:** Association of Alcohol Consumption Patterns between 2015–2016 and 2017–2018 with Depression Risk among Medical Beneficiaries and National Health Insurance beneficiaries

		**Model 1**		*P* value	**Model 2**		*P* value
Alcohol consumption pattern from period 1 to period 2	**Event/total**	**Medical Aid Beneficiaries**^**a**^	**National Health Insurance beneficiaries**^**b**^		**Medical Aid Beneficiaries**^**a**^	**National Health Insurance beneficiaries**^**b**^	
No alcohol consumption	1573/17071	1.43 (1.28–1.60)	1.00 (ref)	< .001	1.37 (1.22–1.54)	1.00 (ref)	< .001
Increased alcohol consumption	316/4766	1.58 (1.24–2.03)	1.00 (ref)	< .001	1.51 (1.17–1.94)	1.00 (ref)	0.002
Decreased alcohol consumption	417/5347	1.53 (1.24–1.90)	1.00 (ref)	< .001	1.48 (1.18–1.84)	1.00 (ref)	< .001
Continuously high alcohol consumption	81/796	2.51 (1.56–4.05)	1.00 (ref)	< .001	2.31 (1.36–3.93)	1.00 (ref)	0.002

The level of alcohol consumption was determined based on the frequency of alcohol intake per week, measured during two consecutive health checkups conducted between 2015–16 (period 1) and 2017–18 (period 2). The frequency pattern between periods was categorized into four groups: (1) no alcohol consumption for both periods, (2) increased alcohol consumption from period 1 to 2, (3) decreased alcohol consumption from period 1 to 2, and (4) continuously high alcohol consumption for both periods (≥ 5 times per week). Depression defines by any antidepressant medication intake or diagnosed by expert physician (ICD-10 F32, F33)

aOR was calculated using multivariate adjusted logistic regression and presented with 95% CI. Event number of depression was presented as n (%). Model 1 was adjusted by age and sex. Model 2 was adjusted by age, sex, household income, baseline comorbidities (hypertension, diabetes, dyslipidemia), cigarette smoking, body mass index, moderate-to-vigorous physical activity, and Charlson Comorbidity Index

^a^Medical Aid Beneficiaries were defined by Participants were selected as Medical Aid Beneficiaries for the first time in their lives between 2017 and 2018

^b^National Health Insurance beneficiaries were define by Participants did not receive medical benefits until 2018

Specifically, across different alcohol consumption groups, the risk of depressive episodes remained significantly higher among medical aid beneficiaries: 2.31 times higher for continuously high alcohol consumers (aOR, 2.31; 95% CI, 1.36–3.93), 1.51 times higher for increased consumption(aOR, 1.51; 95% CI, 1.17–1.94), 1.48 times higher for decreased consumption(aOR, 1.48; 95% CI, 1.18–1.84), and 1.37 times higher for non-consumers(aOR, 1.37; 95% CI, 1.22–1.54). These findings illustrate a persistent elevated risk of depressive episodes among medical aid beneficiaries across varying alcohol consumption levels, indicating a widening gap in depressive episode risks between medical aid beneficiaries and National Health Insurance beneficiaries as alcohol intake patterns change.

### Stratified analysis

The stratified analysis regarding the association between alcohol consumption patterns and the risk of depression incidents is presented in Tables S1 to S4. The analysis stratified age, gender, BMI, smoking status, and CCI into the following four groups: (1) no alcohol consumption for both periods, (2) increased alcohol consumption from period 1 to 2, (3) decreased alcohol consumption from period 1 to 2, and (4) continuously high alcohol consumption for both periods (≥ 5 times per week). Irrespective of alcohol consumption patterns, medical aid beneficiaries consistently exhibited a higher risk of depression compared to National Health Insurance beneficiaries. The stratified analysis based on age, gender, BMI, smoking status, and CCI showed inconsistent odds ratios (ORs) and statistical significance regarding the risk of depression between National Health Insurance beneficiaries and medical aid beneficiaries across the four different alcohol consumption pattern groups (1) to (4). However, in general, individuals aged 65 or older (1.47–2.35 times higher), males (1.48–2.28 times higher), those with a BMI ≥ 25.0 kg/m2 (1.40–1.68 times higher), former or current smokers (1.52–4.30 times higher), and those with a CCI value of ≥ 2 (1.37–2.18 times higher) exhibited higher tendencies for depression incidents among medical aid beneficiaries compared to National Health Insurance beneficiaries, regardless of alcohol consumption patterns.

## Discussion

Regardless of changes in alcohol consumption between the two health check-up periods, it consistently appeared that medical aid beneficiaries had a higher risk of depression compared to National Health Insurance beneficiaries, and these results were generally consistent across the entire subgroup. Our research indicates that moderate drinkers from lower socioeconomic backgrounds face a higher risk of alcohol-related harm compared to the risk attributed solely to alcohol consumption. Furthermore, the study highlighted that among individuals with changes in weekly alcohol intake—specifically, increased or consistently high frequency of intake—the disparity in the risk of depression between the two groups became more pronounced. This suggests that increased alcohol consumption could be one factor contributing to the widening gap in the risk of depression occurrence between medical aid beneficiaries and National Health Insurance beneficiaries.

Our observation of higher depression risk among medical aid beneficiaries compared to National Health Insurance beneficiaries, regardless of changes in alcohol consumption, aligns with established evidence of greater alcohol-related harm in disadvantaged social groups [6, 19, 20]. In a meta-analysis encompassing 133 million individuals, the combined analysis of factors such as education, occupation, employment status, and income revealed that the risk of death from alcohol-related causes is roughly 1.7 times greater than the risk of overall mortality [19]. This emphasizes that while a lower SES is linked to an increased risk of mortality, notably higher risks are specifically associated with deaths attributed to alcohol-related causes [5]. Research findings from analyzing eight cohorts associated with NHS Scotland's Community Health Index indicate that individuals with lower SES and moderate alcohol intake experience greater harm compared to higher SES individuals who consume larger amounts [6]. However, the prospective association between alcohol consumption and depression is complex and not fully understood [21, 22]. Evidence about whether heavy alcohol consumption increases the risk of depression is mixed, with some studies finding a positive association [23, 24], while others reporting no significant effect after adjusting for confounders [25]. Nevertheless, there is limited research exploring the potential influence of socioeconomic status (SES) on the relationship between alcohol consumption and depression. This study suggests that the disparity in depression risk between the two socioeconomic status (SES) groups could be influenced by drinking patterns, such as increased alcohol consumption. However, this doesn't imply that alcohol intake alone accounts for this difference. This is known as the ‘alcohol harm paradox,’ which implies that other factors besides alcohol affect the relationship between SES and alcohol-related outcomes [26].

A comprehensive review involving observational and quantitative studies in the general adult population showed that the quantity consumed per drinking occasion or the frequency of heavy episodic drinking explained 15–30% of socioeconomic inequalities [27]. Overall, this highlights that drinking patterns, not just overall consumption, are crucial in understanding the alcohol-harm paradox [28]. The paradox might be clarified by the 'differential exposure hypothesis,' indicating that lower-SES groups tend to engage in drinking contexts and behaviors—such as binge drinking, public drinking, or simultaneous substance use—that elevate the risk of harm [28, 29]. Alternatively, the differential vulnerability hypothesis suggests that lower-SES groups are more susceptible to the negative effects of alcohol due to their unhealthy lifestyle habits, social support, and coping resources [30, 31], which may all worsen the effects of alcohol consumption on health outcomes, such as depression [32]. The combined findings of a meta-analysis involving ten cross-sectional and longitudinal studies from 2011, along with a recent extensive genomic study utilizing Mendelian randomization, demonstrated a causal relationship supporting problematic alcohol use as a precursor to depression [33]. However, the causal direction between alcohol and depression is unclear, as they may influence each other reciprocally [34]. Moreover, the association between alcohol and depression may vary by socioeconomic status (SES), as lower-SES groups may drink more alcohol as a way of coping with or self-medicating their depression [35]. Our analysis of the NHIS cohort further extends evidence of a stronger association between social and economic inequality and mental health outcomes, especially among individuals with previous high or increasing alcohol consumption patterns, utilizing extensive population-level data with ascertained depression cases.

The strengths of our study include its substantial sample size and the classification of drinking habits, which captures a broad spectrum of drinking behaviors. Moreover, unlike previous studies relying on self-reported measures like the PHQ-9[23], our research managed to investigate the link between alcohol intake and the likelihood of depression across socioeconomic status (SES) using confirmed depression diagnoses within the general population.

### Limitations

While our study provides valuable insights, it has several limitations. First, as our study adopted a cohort study with a nested case–control design, there were limitations in establishing a causal relationship between drinking and depression. Further investigations, encompassing genetic variations, are required to explore the reciprocal association between alcohol consumption and depression. Second, our study has potential limitations due to the predetermined matching method provided by the NHID, which may have introduced bias. Despite our efforts to reduce bias through exact matching and careful exclusion criteria, the complexity of these methods may have inadvertently reinforced selection bias. Third, our study was unable to discern between the types of medical benefits, despite the potential for variations in depression trends between Medical Aid type 1 (intended for individuals unable to work) and Medical Aid type 2 (designed for those capable of working). Fourth, the study lacks external generalizability regarding the association between an individual’s socioeconomic status, alcohol consumption, and depression risk, as the data is restricted to Korea’s Medical Aid beneficiaries and National Health Insurance beneficiaries. The findings may not apply to populations in other countries or to uninsured individuals in Korea. However, to our knowledge, this study stands as the initial research comparing depression risk concerning alcohol consumption among medical aid beneficiaries and that among National Health Insurance beneficiaries, leveraging the comprehensive and representative data from the NHID that encompasses the entire population of South Korea.

## Conclusions

The findings of this study may contribute to the existing literature by highlighting the significance of considering an individual's socioeconomic status when exploring the connection between alcohol consumption and depression. Furthermore, based on the study's findings, it is essential to prioritize public health measures targeting changes in alcohol use, such as previous increases in consumption, to alleviate socioeconomic disparities in alcohol-related depression risks. This emphasis on specific patterns of alcohol consumption may prove more effective in mitigating risks than solely focusing on overall alcohol intake. Nevertheless, further investigation using diverse data sources is crucial to comprehensively understand the alcohol-related harm paradox. Longitudinal studies are necessary to explore the temporal relationship between alcohol and depression across various socioeconomic groups.

### Supplementary Information

Supplementary Material 1

## Data Availability

The study findings are supported by data from the Korea NHIS, but access to these data is restricted and used under license for this current study, thus not publicly accessible. Nonetheless, the authors* can provide the data upon reasonable request and with permission from the Korean NHIS. * Su Kyoung Lee, email: susu2062@gmail.com; Yong Jin Kwon, Email: mirae@snu.ac.kr.
