# Supplementary material for: Socioeconomic disparities in alcohol-related depression: a national cohort study of low-income medical aid beneficiaries and national health insurance beneficiaries in Korea
Source: BMC Public Health. 2024 Aug 13;24:2189. doi: 10.1186/s12889-024-19665-6 (PMC11321173; doi:10.1186/s12889-024-19665-6)
Supplement: Supplementary file 1 — Supplementary Material 1 [file 12889_2024_19665_MOESM1_ESM.docx]

**Supplementary Materials for**

**“Title”**

**Author**

**Table of content**

**Supplementary table 1. Subgroup analysis for no alcohol consumption from period 1 and 2**

**Supplementary table 2. Subgroup analysis for increased alcohol consumption from period 1 and 2**

**Supplementary table 3. Subgroup analysis for decreased alcohol consumption from period 1 and 2**

**Supplementary table 4. Subgroup analysis for continuously heavy drinking from period 1 and 2**

**Supplementary table 5. Association of Changes in Alcohol Consumption with the Risk of Depression (Categorized by Weekly Alcohol Consumption Frequency)**

**Supplementary Table 6. The association between the amount of alcohol consumption and depression events**

**Supplementary Table 7. Association of Changes in Alcohol Consumption with Depression Risk among Medical Benefit Recipients and Health Insurance Subscribers using survival analysis**

**Supplementary Table 8. Association of Alcohol Consumption Patterns between 2015-2016 and 2017-2018 with Depression Risk among Medical Beneficiaries and Health Insurance Subscribers using survival analysis**

**Supplementary table 1. Subgroup analysis for no alcohol consumption from period 1 and 2**

| Variables | **Multivariable-adjusted OR (95% CI)** | | P value | P for interaction |
| --- | --- | --- | --- | --- |
|  | **Medical Benefit Recipients^a^** | **Health Insurance Subscribers^b^** |  |  |
| **Age** | | | | 0.49 |
| ≥ 65 years | 1.47 (1.24-1.73) | 1.00 (ref) | <.001 |  |
| < 65 years | 1.29 (1.09-1.53) | 1.00 (ref) | 0.003 |  |
| **Sex** | | |  | 0.27 |
| Male | 1.48 (1.20-1.83) | 1.00 (ref) | <.001 |  |
| Female | 1.31 (1.14-1.51) | 1.00 (ref) | <.001 |  |
| **Body mass index** | | | | 0.31 |
| <18.5 kg/m2 | 0.94 (0.43-2.04) | 1.00 (ref) | 0.87 |  |
| 18.5-23.0 kg/m2 | 1.28 (1.05-1.58) | 1.00 (ref) | 0.02 |  |
| 23.0-25.0 kg/m2 | 1.50 (1.19-1.89) | 1.00 (ref) | <.001 |  |
| ≥25.0 kg/m2 | 1.40 (1.16-1.68) | 1.00 (ref) | <.001 |  |
| **Cigarette smoking** | | | | 0.18 |
| Non-smoker | 1.32 (1.16-1.51) | 1.00 (ref) | <.001 |  |
| Former-smoker | 1.52 (1.09-2.14) | 1.00 (ref) | 0.02 |  |
| Current smoker | 1.61 (1.08-2.39) | 1.00 (ref) | 0.02 |  |
| **Charlson comorbidity index** | | | | 0.98 |
| 0 | 1.36 (1.12-1.65) | 1.00 (ref) | 0.002 |  |
| 1 | 1.40 (1.13-1.73) | 1.00 (ref) | 0.002 |  |
| ≥2 | 1.37 (1.11-1.68) | 1.00 (ref) | 0.003 |  |

The level of alcohol consumption was determined based on the frequency of alcohol intake per week, measured during two consecutive health checkups conducted between 2015-16 (period 1) and 2017-18 (period 2). The frequency pattern between periods was categorized into four groups: (1) no alcohol consumption for both periods, (2) increased alcohol consumption from period 1 to 2, (3) decreased alcohol consumption from period 1 to 2, and (4) continuously high alcohol consumption for both periods (≥5 times per week).

Depression defines by any antidepressant medication intake or diagnosed by expert physician (ICD-10 F32, F33)

aOR was calculated using multivariate adjusted logistic regression and presented with 95% CI. Adjusted by age, sex, household income, baseline comorbidities (hypertension, diabetes, dyslipidemia), cigarette smoking, body mass index, moderate-to-vigorous physical activity, and Charlson Comorbidity Index.

^a^Medical Benefit Recipients were defined by Participants were selected as Medical Benefit Recipients for the first time in their lives between 2017 and 2018.

^b^Health Insurance Subscribers were define by Participants did not receive medical benefits until 2018

**Supplementary table 2. Subgroup analysis for increased alcohol consumption from period 1 and 2**

| Variables | **Multivariable-adjusted OR (95% CI)** | | P value | P for interaction |
| --- | --- | --- | --- | --- |
|  | **Medical Benefit Recipients^a^** | **Health Insurance Subscribers^b^** |  |  |
| **Age** | | | | 0.72 |
| ≥ 65 years | 1.57 (1.01-2.43) | 1.00 (ref) | 0.04 |  |
| < 65 years | 1.53 (1.12-2.11) | 1.00 (ref) | 0.009 |  |
| **Sex** | | | | 0.89 |
| Male | 1.52 (1.12-2.06) | 1.00 (ref) | 0.007 |  |
| Female | 1.51 (0.93-2.44) | 1.00 (ref) | 0.10 |  |
| **Body mass index** | | | | 0.83 |
| <18.5 kg/m2 | 1.50 (0.11-20.23) | 1.00 (ref) | 0.89 |  |
| 18.5-23.0 kg/m2 | 1.35 (0.86-2.13) | 1.00 (ref) | 0.19 |  |
| 23.0-25.0 kg/m2 | 1.42 (0.87-2.31) | 1.00 (ref) | 0.16 |  |
| ≥25.0 kg/m2 | 1.68 (1.10-2.56) | 1.00 (ref) | 0.02 |  |
| **Cigarette smoking** | | | | 0.28 |
| Non-smoker | 1.36 (0.92-2.01) | 1.00 (ref) | 0.12 |  |
| Former-smoker | 1.45 (0.88-2.41) | 1.00 (ref) | 0.15 |  |
| Current smoker | 1.90 (1.17-3.10) | 1.00 (ref) | 0.10 |  |
| **Charlson comorbidity index** | | | | 0.66 |
| 0 | 1.74 (1.17-2.58) | 1.00 (ref) | 0.006 |  |
| 1 | 1.08 (0.65-1.78) | 1.00 (ref) | 0.77 |  |
| ≥2 | 1.64 (1.02-2.64) | 1.00 (ref) | 0.04 |  |

The level of alcohol consumption was determined based on the frequency of alcohol intake per week, measured during two consecutive health checkups conducted between 2015-16 (period 1) and 2017-18 (period 2). The frequency pattern between periods was categorized into four groups: (1) no alcohol consumption for both periods, (2) increased alcohol consumption from period 1 to 2, (3) decreased alcohol consumption from period 1 to 2, and (4) continuously high alcohol consumption for both periods (≥5 times per week).

Depression defines by any antidepressant medication intake or diagnosed by expert physician (ICD-10 F32, F33)

aOR was calculated using multivariate adjusted logistic regression and presented with 95% CI. Adjusted by age, sex, household income, baseline comorbidities (hypertension, diabetes, dyslipidemia), cigarette smoking, body mass index, moderate-to-vigorous physical activity, and Charlson Comorbidity Index.

^a^Medical Benefit Recipients were defined by Participants were selected as Medical Benefit Recipients for the first time in their lives between 2017 and 2018.

^b^Health Insurance Subscribers were define by Participants did not receive medical benefits until 2018

**Supplementary table 3. Subgroup analysis for decreased alcohol consumption from period 1 and 2**

| Variables | **Multivariable-adjusted OR (95% CI)** | | P value | P for interaction |
| --- | --- | --- | --- | --- |
|  | **Medical Benefit Recipients^a^** | **Health Insurance Subscribers^b^** |  |  |
| **Age** | | | | 0.11 |
| ≥ 65 years | 2.02 (1.38-2.95) | 1.00 (ref) | <.001 |  |
| < 65 years | 1.26 (0.95-1.66) | 1.00 (ref) | 0.11 |  |
| **Sex** | | | | 0.92 |
| Male | 1.49 (1.14-1.93) | 1.00 (ref) | 0.003 |  |
| Female | 1.42 (0.94-2.16) | 1.00 (ref) | 0.10 |  |
| **Body mass index** | | | | 0.50 |
| <18.5 kg/m2 | 0.55 (0.10-3.12) | 1.00 (ref) | 0.50 |  |
| 18.5-23.0 kg/m2 | 1.58 (1.06-2.35) | 1.00 (ref) | 0.03 |  |
| 23.0-25.0 kg/m2 | 1.37 (0.86-2.19) | 1.00 (ref) | 0.19 |  |
| ≥25.0 kg/m2 | 1.56 (1.11-2.19) | 1.00 (ref) | 0.01 |  |
| **Cigarette smoking** | | | | 0.96 |
| Non-smoker | 1.45 (1.05-2.00) | 1.00 (ref) | 0.03 |  |
| Former-smoker | 1.55 (1.00-2.41) | 1.00 (ref) | 0.05 |  |
| Current smoker | 1.51 (0.98-2.33) | 1.00 (ref) | 0.06 |  |
| **Charlson comorbidity index** | | | | 0.03 |
| 0 | 1.06 (0.74-1.53) | 1.00 (ref) | 0.76 |  |
| 1 | 1.70 (1.11-2.58) | 1.00 (ref) | 0.01 |  |
| ≥2 | 2.18 (1.45-3.27) | 1.00 (ref) | <.001 |  |

The level of alcohol consumption was determined based on the frequency of alcohol intake per week, measured during two consecutive health checkups conducted between 2015-16 (period 1) and 2017-18 (period 2). The frequency pattern between periods was categorized into four groups: (1) no alcohol consumption for both periods, (2) increased alcohol consumption from period 1 to 2, (3) decreased alcohol consumption from period 1 to 2, and (4) continuously high alcohol consumption for both periods (≥5 times per week).

Depression defines by any antidepressant medication intake or diagnosed by expert physician (ICD-10 F32, F33)

aOR was calculated using multivariate adjusted logistic regression and presented with 95% CI. Adjusted by age, sex, household income, baseline comorbidities (hypertension, diabetes, dyslipidemia), cigarette smoking, body mass index, moderate-to-vigorous physical activity, and Charlson Comorbidity Index.

^a^Medical Benefit Recipients were defined by Participants were selected as Medical Benefit Recipients for the first time in their lives between 2017 and 2018.

^b^Health Insurance Subscribers were define by Participants did not receive medical benefits until 2018

**Supplementary table 4. Subgroup analysis for continuously heavy drinking from period 1 and 2**

| **Variables** | **Multivariable-adjusted OR (95% CI)** | | P value | P for interaction |
| --- | --- | --- | --- | --- |
|  | **Medical Benefit Recipients^a^** | **Health Insurance Subscribers^b^** |  |  |
| **Age** | | | | 0.81 |
| ≥ 65 years | 2.35 (1.06-5.24) | 1.00 (ref) | 0.04 |  |
| < 65 years | 2.30 (1.08-4.91) | 1.00 (ref) | 0.03 |  |
| **Sex** | | | | 0.98 |
| Male | 2.28 (1.29-4.02) | 1.00 (ref) | 0.004 |  |
| Female | NA | 1.00 (ref) | NA |  |
| **Body mass index** | | | | 0.54 |
| <18.5 kg/m2 | NA | 1.00 (ref) | NA |  |
| 18.5-23.0 kg/m2 | 3.89 (1.36-11.16) | 1.00 (ref) | 0.01 |  |
| 23.0-25.0 kg/m2 | 3.11 (0.85-11.29) | 1.00 (ref) | 0.09 |  |
| ≥25.0 kg/m2 | 2.22 (0.96-5.14) | 1.00 (ref) | 0.06 |  |
| **Cigarette smoking** | | | | 0.32 |
| Non-smoker | 3.60 (0.84-15.45) | 1.00 (ref) | 0.08 |  |
| Former-smoker | 1.64 (0.68-3.94) | 1.00 (ref) | 0.27 |  |
| Current smoker | 4.30 (1.67-11.04) | 1.00 (ref) | 0.003 |  |
| **Charlson comorbidity index** | | | | 0.02 |
| 0 | 4.21 (1.45-12.20) | 1.00 (ref) | 0.008 |  |
| 1 | 1.72 (0.68-4.36) | 1.00 (ref) | 0.26 |  |
| ≥2 | 2.24 (0.87-5.80) | 1.00 (ref) | 0.10 |  |

The level of alcohol consumption was determined based on the frequency of alcohol intake per week, measured during two consecutive health checkups conducted between 2015-16 (period 1) and 2017-18 (period 2). The frequency pattern between periods was categorized into four groups: (1) no alcohol consumption for both periods, (2) increased alcohol consumption from period 1 to 2, (3) decreased alcohol consumption from period 1 to 2, and (4) continuously high alcohol consumption for both periods (≥5 times per week).

Depression defines by any antidepressant medication intake or diagnosed by expert physician (ICD-10 F32, F33)

aOR was calculated using multivariate adjusted logistic regression and presented with 95% CI. Adjusted by age, sex, household income, baseline comorbidities (hypertension, diabetes, dyslipidemia), cigarette smoking, body mass index, moderate-to-vigorous physical activity, and Charlson Comorbidity Index.

^a^Medical Benefit Recipients were defined by Participants were selected as Medical Benefit Recipients for the first time in their lives between 2017 and 2018.

^b^Health Insurance Subscribers were define by Participants did not receive medical benefits until 2018

**Supplementary table 5. Association of Changes in Alcohol Consumption with the Risk of Depression (Categorized by Weekly Alcohol Consumption Frequency)**

|  | **Event/total** | **Multivariable-adjusted OR (95% CI)** | | P value |
| --- | --- | --- | --- | --- |
|  |  | **Medical Benefit Recipients^a^** | **Health Insurance Subscribers^b^** |  |
| **Increased alcohol consumption** | | | | |
| 0 days/week (start point) | 178/2608 | 1.53 (1.09-2.15) | 1.00 (ref) | 0.01 |
| 1-2 days/week | 107/1735 | 1.88 (1.22-2.90) | 1.00 (ref) | 0.004 |
| 3-4 days/week | 31/423 | 0.77 (0.29-2.03) | 1.00 (ref) | 0.59 |
| **Decreased alcohol consumption** | | | | |
| ≥5 days/week (start point) | 72/798 | 1.86 (1.08-3.19) | 1.00 (ref) | 0.02 |
| 3-4 days/week | 124/1754 | 1.67 (1.11-2.51) | 1.00 (ref) | 0.01 |
| 1-2 days/week | 221/2795 | 1.35 (0.99-1.84) | 1.00 (ref) | 0.06 |

The level of alcohol consumption was determined based on the frequency of alcohol intake per week, measured during two consecutive health checkups conducted between 2015-16 (period 1) and 2017-18 (period 2). Increased alcohol consumption from period 1 to 2, Decreased alcohol consumption from period 1 to 2.

Depression defines by any antidepressant medication intake or diagnosed by expert physician (ICD-10 F32, F33)

aOR was calculated using multivariate adjusted logistic regression and presented with 95% CI. Adjusted by age, sex, household income, baseline comorbidities (hypertension, diabetes, dyslipidemia), cigarette smoking, body mass index, moderate-to-vigorous physical activity, and Charlson Comorbidity Index. Event number of depression was presented as n (%).

^a^Medical Benefit Recipients were defined by Participants were selected as Medical Benefit Recipients for the first time in their lives between 2017 and 2018.

^b^Health Insurance Subscribers were define by Participants did not receive medical benefits until 2018

**Supplementary Table 6. The association between the amount of alcohol consumption and depression events**

|  | **Multivariable-adjusted OR (95% CI)** | | P value |
| --- | --- | --- | --- |
|  | **Medical Benefit Recipients^a^** | **Health Insurance Subscribers^b^** |  |
| **Alcohol consumption by standard drinks per week**  **Period 1 (2015-16)** | | |  |
| 0 standard drinks | 1.20 (0.88-1.62) | 1.00 (ref) | 0.25 |
| 1-5 standard drinks | 0.92 (0.68-1.23) | 1.00 (ref) | 0.57 |
| 6-9 standard drinks | 1.17 (0.79-1.75) | 1.00 (ref) | 0.43 |
| ≥ 10 standard drinks | 1.34 (1.07-1.69) | 1.00 (ref) | 0.01 |
| **Alcohol consumption by standard drinks per week**  **Period 2 (2017)** | | |  |
| 0 standard drinks | 1.14 (0.89-1.45) | 1.00 (ref) | 0.29 |
| 1-5 standard drinks | 1.22 (0.92-1.62) | 1.00 (ref) | 0.16 |
| 6-9 standard drinks | 0.98 (0.66-1.46) | 1.00 (ref) | 0.93 |
| ≥ 10 standard drinks | 1.28 (0.96-1.72) | 1.00 (ref) | 0.10 |
| **Alcohol consumption pattern by standard drinks** | | |  |
| No alcohol consumption from period 1 and 2 | 1.19 (0.87-1.63) | 1.00 (ref) | 0.29 |
| Increased alcohol consumption from period 1 to 2 | 1.21 (0.75-1.94) | 1.00 (ref) | 0.44 |
| Decreased alcohol consumption from period 1 to 2 | 1.26 (1.00-1.60) | 1.00 (ref) | 0.05 |
| Continuously high alcohol consumption for period 1 and 2 | 1.24 (0.89-1.72) | 1.00 (ref) | 0.20 |

The amount of alcohol consumption was calculated by multiplying the frequency and quantity of alcohol consumed per week. Each participant reported their alcohol frequency from 0 to 7 days per week and the amount from 0 to 100 standard drinks, irrespective of the alcohol type. Subsequently, total alcohol consumption in standard drinks was divided into four categories: 0, 1-5, 6-9, and ≥10 standard drinks for both 2015-16 (period 1) and 2017-18 (period 2).

Comparatively, alcohol consumption in standard drinks was categorized into four groups based on the change from period 1 to period 2: (1) No alcohol consumption in either period, (2) Increased consumption from period 1 to 2, (3) Decreased consumption from period 1 to 2, (4) Consistently high consumption across both periods. Only participants who underwent health screenings in 2017 were included in the analysis due to changes in alcohol consumption questionnaires between health screenings in 2018.

Depression defines by any antidepressant medication intake or diagnosed by expert physician (ICD-10 F32, F33)

aOR was calculated using multivariate adjusted logistic regression and presented with 95% CI. Adjusted by age, sex, household income, baseline comorbidities (hypertension, diabetes, dyslipidemia), cigarette smoking, body mass index, moderate-to-vigorous physical activity, and Charlson Comorbidity Index. Event number of depression was presented as n (%).

^a^Medical Benefit Recipients were defined by Participants were selected as Medical Benefit Recipients for the first time in their lives between 2017 and 2018.

^b^Health Insurance Subscribers were define by Participants did not receive medical benefits until 2018

**Supplementary Table 7. Association of Changes in Alcohol Consumption with Depression Risk among Medical Benefit Recipients and Health Insurance Subscribers using survival analysis**

| **Alcohol consumption per week** | **Multivariable-adjusted HR (95% CI)** | | P value |
| --- | --- | --- | --- |
|  | **Medical Benefit Recipients^a^** | **Health Insurance Subscribers^b^** |  |
| **Period 1 (2015-16)** | | | |
| 0 days | 1.37 (1.23-1.52) | 1.00 (ref) | <.001 |
| 1-2 days | 1.35 (1.16-1.58) | 1.00 (ref) | <.001 |
| 3-4 days | 1.42 (1.09-1.85) | 1.00 (ref) | 0.009 |
| ≥ 5 days | 1.84 (1.30-2.60) | 1.00 (ref) | <.001 |
| **Period 2 (2017-18)** | | | |
| 0 days | 1.37 (1.23-1.51) | 1.00 (ref) | <.001 |
| 1-2 days | 1.37 (1.17-1.61) | 1.00 (ref) | <.001 |
| 3-4 days | 1.39 (1.05-1.83) | 1.00 (ref) | 0.02 |
| ≥ 5 days | 1.89 (1.34-2.67) | 1.00 (ref) | <.001 |

The level of alcohol consumption was determined based on the frequency of alcohol intake per week, measured during two consecutive health checkups conducted between 2015-16 (period 1) and 2017-18 (period 2). Depression defines by any antidepressant medication intake or diagnosed by expert physician (ICD-10 F32, F33)

aHR was calculated using multivariate adjusted Cox regression and presented with 95% CI. Adjusted by age, sex, household income, baseline comorbidities (hypertension, diabetes, dyslipidemia), cigarette smoking, body mass index, moderate-to-vigorous physical activity, and Charlson Comorbidity Index.

^a^Medical Benefit Recipients were defined by Participants were selected as Medical Benefit Recipients for the first time in their lives between 2017 and 2018.

^b^Health Insurance Subscribers were define by Participants did not receive medical benefits until 2018

**Supplementary Table 8. Association of Alcohol Consumption Patterns between 2015-2016 and 2017-2018 with Depression Risk among Medical Beneficiaries and Health Insurance Subscribers using survival analysis**

|  |  | **Model 1** | | P value | **Model 2** | | P value |
| --- | --- | --- | --- | --- | --- | --- | --- |
| **Alcohol consumption pattern from period 1 to period 2** | **Event/total** | **Medical Benefit Recipients^a^** | **Health Insurance Subscribers^b^** |  | **Medical Benefit Recipients^a^** | **Health Insurance Subscribers^b^** |  |
| **No alcohol consumption** | 1573/17071 | 1.41 (1.27-1.57) | 1.00 (ref) | <.001 | 1.35 (1.21-1.51) | 1.00 (ref) | <.001 |
| **Increased alcohol consumption** | 316/4766 | 1.41 (1.22-1.62) | 1.00 (ref) | <.001 | 1.38 (1.19-1.59) | 1.00 (ref) | <.001 |
| **Decreased alcohol consumption** | 417/5347 | 1.52 (1.24-1.86) | 1.00 (ref) | <.001 | 1.46 (1.19-1.80) | 1.00 (ref) | <.001 |
| **Continuously high alcohol consumption** | 81/796 | 2.30 (1.48-3.57) | 1.00 (ref) | <.001 | 2.04 (1.26-3.30) | 1.00 (ref) | 0.004 |

The level of alcohol consumption was determined based on the frequency of alcohol intake per week, measured during two consecutive health checkups conducted between 2015-16 (period 1) and 2017-18 (period 2). The frequency pattern between periods was categorized into four groups: (1) no alcohol consumption for both periods, (2) increased alcohol consumption from period 1 to 2, (3) decreased alcohol consumption from period 1 to 2, and (4) continuously high alcohol consumption for both periods (≥5 times per week). Depression defines by any antidepressant medication intake or diagnosed by expert physician (ICD-10 F32, F33).

aHR was calculated using multivariate adjusted Cox regression and presented with 95% CI. Event number of depression was presented as n (%). Model 1 was adjusted by age and sex. Model 2 was adjusted by age, sex, household income, baseline comorbidities (hypertension, diabetes, dyslipidemia), cigarette smoking, body mass index, moderate-to-vigorous physical activity, and Charlson Comorbidity Index.

^a^Medical Benefit Recipients were defined by Participants were selected as Medical Benefit Recipients for the first time in their lives between 2017 and 2018.

^b^Health Insurance Subscribers were define by Participants did not receive medical benefits until 2018
